# Diagnostic impact of [^18^F]flutemetamol PET in early-onset dementia

**DOI:** 10.1186/s13195-016-0228-4

**Published:** 2017-01-17

**Authors:** Marissa D. Zwan, Femke H. Bouwman, Elles Konijnenberg, Wiesje M. van der Flier, Adriaan A. Lammertsma, Frans R. J. Verhey, Pauline Aalten, Bart N. M. van Berckel, Philip Scheltens

**Affiliations:** 1 Alzheimer Center & Department of Neurology, Amsterdam Neuroscience, VU University Medical Center, PO Box 7057, 1007 MB Amsterdam, The Netherlands; 2 Department of Radiology & Nuclear Medicine, Amsterdam Neuroscience, VU University Medical Center, Amsterdam, The Netherlands; 3 Department of Epidemiology & Biostatistics, Amsterdam Neuroscience, VU University Medical Center, Amsterdam, The Netherlands; 4 School for Mental Health and Neuroscience, Alzheimer Centre Limburg, European Graduate School of Neuroscience EURON, Maastricht University, Maastricht, The Netherlands

**Keywords:** Alzheimer’s disease, Dementia, Clinical practice, Diagnostic impact, Positron emission tomography, Imaging, Amyloid

## Abstract

**Background:**

Early-onset dementia patients often present with atypical clinical symptoms, hampering an accurate clinical diagnosis. The purpose of the present study was to assess the diagnostic impact of the amyloid-positron emission tomography (PET) imaging agent [^18^F]flutemetamol in early-onset dementia patients, in terms of change in (confidence in) diagnosis and patient management plan.

**Methods:**

This prospective bi-center study included 211 patients suspected of early-onset dementia who visited a tertiary memory clinic. Patients were eligible with Mini Mental State Examination ≥ 18 and age at diagnosis ≤ 70 years and in whom the diagnostic confidence was <90% after routine diagnostic work-up. All patients underwent [^18^F]flutemetamol PET, which was interpreted as amyloid-negative or amyloid-positive based on visual rating. Before and after disclosing the PET results, we assessed the diagnostic confidence (using a visual analog scale of 0–100%) and clinical diagnosis. The impact of [^18^F]flutemetamol PET on the patient management plan was also evaluated.

**Results:**

[^18^F]flutemetamol PET scans were positive in 133 out of 211 (63%) patients, of whom 110 out of 144 (76%) patients had a pre-PET Alzheimer’s disease (AD) diagnosis and 23 out of 67 (34%) patients had a non-AD diagnosis. After disclosure of PET results, 41/211 (19%) diagnoses changed. Overall, diagnostic confidence increased from 69 ± 12% to 88 ± 15% after disclosing PET results (*P* < 0.001; in 87% of patients). In 79 (37%) patients, PET results led to a change in patient management and predominantly the initiation of AD medication when PET showed evidence for amyloid pathology.

**Conclusions:**

[^18^F]flutemetamol PET changed clinical diagnosis, increased overall diagnostic confidence, and altered the patient management plan. Our results suggest that amyloid PET may have added value over the standardized diagnostic work-up in early-onset dementia patients with uncertain clinical diagnosis. This study provides evidence for the recommendations put forward in the appropriate use criteria for amyloid PET in clinical practice.

**Trial registration:**

Nederlands Trial Register NTR3743. Registered 7 December 2012.

## Background

In patients suspected of early-onset dementia, accurate clinical diagnosis may be challenging. Patients with early-onset Alzheimer’s disease (AD) more often present with atypical clinical symptoms, such as difficulties with vision or speech, behavioral changes, or problems with handling tools, compared with older AD patients who typically present with memory problems [1, 2]. These atypical clinical symptoms often overlap with symptoms of other early-onset dementia types, hampering an accurate clinical diagnosis necessary for both prognosis and treatment.

Several fluorine-18-labeled positron emission tomography (PET) tracers, including [^18^F]flutemetamol, have become available for clinical practice and incorporated as amyloid pathology biomarkers in the revised research criteria for AD [3]. In addition, criteria for the appropriate use of amyloid PET state a potential added value of amyloid PET in diagnosing patients with (persistent or unexplained) MCI or possible AD with unclear clinical presentation and/or young onset of disease [4]; however, at the time of publication of these criteria only little evidence was available and no empirical studies were published. To date, only a few studies have evaluated the effect of amyloid PET on clinical diagnosis and patient management, which were performed in small or highly selected research populations or in combination with [^18^F]fluorodeoxyglucose ([^18^F]FDG) PET [5–11].

In our previous study we assessed the diagnostic value of PET tracers [^11^C]Pittsburgh compound B ([^11^C]PiB) and [^18^F]FDG to detect cortical amyloid deposition and hypometabolic patterns in an unselected tertiary memory clinic [6]. Our findings indicated that amyloid PET changed clinical diagnosis only when diagnostic confidence was <90% and predominantly in (mildly) demented patients. The present cohort study included young-onset and mildly demented patients visiting two Dutch tertiary memory clinics. The aim was to assess the impact of [^18^F]flutemetamol PET on (confidence in) clinical diagnosis and patient management plan.

## Methods

### Patients

The present study included a consecutive series of patients visiting a Dutch tertiary memory clinic and suspected of mild dementia (defined as Mini Mental State Examination (MMSE) score ≥ 18) or early-onset dementia (defined by age at diagnosis ≤ 70 years), who had no firm diagnosis after the standardized dementia evaluation or persisting diagnostic uncertainty (defined as pre-PET diagnostic confidence < 90% as measured by a standardized study questionnaire). We excluded 17 dementia patients with MMSE ≥ 18 and age at diagnosis ≤ 70 years because diagnostic confidence after standardized work-up was lower than 90% (cut-off based on findings in our previous study) [6].

We included 211 patients, of whom 200 patients were recruited from the VU University Medical Center as part of the Amsterdam Dementia Cohort [12] and 11 patients were recruited from the Maastricht University Medical Center. All patients received a standard dementia evaluation that included medical history, informant-based history, physical and neurological examinations, screening laboratory tests, brain magnetic resonance imaging (MRI), and neuropsychological testing. In addition, in the absence of contraindications, a lumbar puncture was performed. For the purpose of this study, lumbar puncture results were not disclosed before the impact of PET results had been assessed. Clinical diagnosis was established by consensus in a multidisciplinary meeting using established clinical criteria [13–17] without knowledge of PET or CSF results or APOE carrier status. Patients were divided into groups based on expected underlying etiology: AD, frontotemporal dementia (FTD), other dementia diagnosis (OD), and non-neurodegenerative diagnosis (NN). More specifically, the AD group consisted of 138 AD patients and six patients with logopenic-variant primary progressive aphasia (lv-PPA); the FTD group consisted of 20 patients with behavioral-variant frontotemporal dementia (bvFTD), six patients with primary nonfluent aphasia (PNFA), and two patients with semantic dementia (SD); the OD group consisted of seven patients with dementia with Lewy bodies (DLB), four patients with corticobasal syndrome (CBS), four patients with progressive supranuclear palsy (PSP), and three patients with vascular dementia (VaD); and the NN group consisted of 12 patients with a psychiatric diagnosis, three patients with chronic traumatic encephalopathy (CTE), two patients with meningeoma, one patient with post-traumatic stress syndrome, one patient with obstructive sleep apnea syndrome (OSAS), and one patient with limbic encephalitis. This study was approved by the medical ethics review committee of the VU University Medical Center (reference number 2012/302).

### Assessment of diagnostic impact

During a multidisciplinary meeting, at which the initial clinical diagnosis was made (and prior to PET), the local study physician (FHB or FRJV) indicated the most probable and differential etiological diagnosis using a questionnaire and estimated their level of diagnostic confidence on a visual analog scale from 0 to 100% for the most probable diagnosis. It was mandatory for the neurologist to make a diagnosis. After PET results were disclosed, clinicians completed the second questionnaire again including a re-evaluation of the (etiological) diagnosis and estimation of diagnostic confidence. In addition, taking into account the PET results, requests for ancillary investigations (e.g., [^18^F]FDG PET scan, lumbar puncture, DaT scan, lumbar puncture, consult other specialist, laboratory tests), initiation or withdrawal of AD medication (e.g., cholinesterase inhibitors, memantine, Souvenaid®), and initiation or withdrawal of relevant care (case manager, day care, speech therapy) were reported. Finally, changes in clinical diagnosis and the patient management plan after disclosure of PET results were verified with hospital medical records.

The mean interval between dementia evaluation and [^18^F]flutemetamol PET scan was 71 ± 136 days. When PET results were disclosed, the neurologist responsible for the initial diagnosis re-evaluated the most probable diagnosis with corresponding diagnostic confidence and patient management plan, now taking into account the PET results. Between baseline dementia evaluation and disclosure of PET results, no other diagnostic test results were disclosed to the neurologist.

### PET scan and interpretation

In both centers, [^18^F]flutemetamol PET scans were made on a Gemini TF-64 PET/CT scanner (Philips Medical Systems, Best, the Netherlands) [18]. Ninety minutes after a bolus injection of 191 ± 10 MBq [^18^F]flutemetamol, patients underwent a low-dose CT scan followed by a 20-minute (i.e., 4 frames of 5 minutes) PET scan. Scans were checked for movement and frames were summed to obtain a static (20-minute) image for each patient (except for one patient in whom the last frame was not used due to extensive head movement). Scans were visually assessed and dichotomously rated as either amyloid-positive or amyloid-negative by the local nuclear medicine physician, who completed the training program for visual interpretation of [^18^F]flutemetamol images. Readers were blinded to clinical information, except for brain MRI.

### Statistical analysis

Differences in baseline characteristics between diagnostic groups were assessed using analysis of variance, Kruskal–Wallis tests, and Pearson χ^2^ tests where appropriate. Clinical dementia rating (CDR) was not available for 11 AD patients, two OD patients, and two NN patients; APOE genotyping was not performed in 15 AD patients, three FTD patients, two OD patients, and three NN patients. Differences in diagnostic confidence prior to PET between clinical diagnoses were assessed using ANOVAs. Change in diagnostic confidence after PET was assessed using paired-sample *t* tests. Pearson χ^2^ tests were used to assess differences in the patient management plan. Association of diagnostic confidence prior to PET with proportion of changed diagnosis and proportion of changed management plan was calculated using linear-by-linear χ^2^. The level of significance was set at *P* < 0.05.

## Results

### Patients

Patients’ demographic and clinical characteristics are presented in Table 1. Overall, the age of the patients was 62 ± 6 years, 45% (*n* = 95) were female, and MMSE was 23 ± 4. In 27 out of 144 (19%) patients with an AD diagnosis prior to PET, AD medication was already prescribed prior to PET.

**Table 1:** Demographic and clinical characteristics according to clinical diagnosis prior to [^18^F]flutemetamol PET

Pre-PET etiology	AD (*n* = 144)	FTD (*n* = 28)	OD (*n* = 19)	NN (*n* = 20)
Age (years)	62 ± 6 (45–70)	62 ± 5 (52–69)	63 ± 6 (48–69)	60 ± 5 (49–69)
Gender, female	71 (49%)	13 (46%)	7 (37%)	4 (20%)
MMSE	23 ± 3	25 ± 3	24 ± 4	24 ± 4
CDR (0.5/1.0/2.0)	77/50/6	18/9/1	8/9/0	13/4/1
APOE genotype, e4 carrier	87 (67%)	7 (28%)^a^	10 (63%)	14 (78%)
Specified diagnosis	6 lv-PPA 138 AD	20 bvFTD 2 SD 6 PNFA	3 VaD 7 DLB 5 CBD 4 PSP	12 psychiatry 3 CTE 2 meningeoma 1 PTSS 1 OSAS 1 limbic encephalitis

Data are presented as mean ± SD (range), *n* (%), or mean ± SD unless stated otherwise. Differences between groups were assessed using ANOVA with post-hoc Bonferroni tests (age and MMSE), χ^2^ tests (gender, APOE genotype), and Kruskal–Wallis with post-hoc Mann–Whitney *U* tests (CDR)

^a^FTD < other diagnostic groups; *P* < 0.05

*PET* positron emission tomography, *MMSE* Mini Mental State Examination, *CDR* clinical dementia rating. *AD* Alzheimer’s disease dementia, *lv-PPA* logopenic-variant primary progressive aphasia, *FTD* frontotemporal dementia, *bvFTD* behavioral variant FTD, *SD* semantic dementia, *PNFA*, primary nonfluent aphasia, *OD* other dementia diagnosis, *NN* non-neurodegenerative diagnosis, *VaD* vascular dementia *DLB* dementia with Lewy bodies, *CBD* corticobasal degeneration, *PSP* progressive supranuclear palsy, *CTE* chronic traumatic encephalopathy, *PTSS* posttraumatic stress syndrome, *OSAS* obstructive sleep apnea syndrome

### Clinical diagnosis

In 59 (28%) patients, the PET findings were inconsistent with expected PET results prior to scanning. This resulted in a change in diagnosis after disclosing PET results in 41 patients (19%).

Table 2 presents an overview of clinical diagnoses before and after disclosing PET results. In patients with an initial AD diagnosis, 111 out of 145 (77%) patients had a positive PET scan. A negative PET scan in patients with an initial diagnosis of AD led to a change in diagnosis in 26 out of 34 (76%) patients. In the remaining 8 (24%) patients with phenotypic AD, the clinical diagnosis remained unchanged after PET results were found to be amyloid-negative.

**Table 2:** Impact of [^18^F]flutemetamol PET on clinical diagnosis according to clinical diagnosis prior to PET

Pre-PET etiology	AD (*n* = 144)		FTD (*n* = 28)		OD (*n* = 19)		NN (*n* = 20)	
PET result	Positive	Negative	Positive	Negative	Positive	Negative	Positive	Negative
*n*	110	34	6	22	8	11	9	11
Change in diagnosis	0 (0%)	26 (76%)	4 (67%)	0 (0%)	2 (25%)	0 (0%)	9 (100%)	0 (0%)
Changed diagnosis after PET		12 NN 7 FTD 3 DLB 2 CBD 1 VaD 1 CTE	4 AD		1 DLB 1 AD		9 AD	
Pre-PET diagnostic confidence (%)	72 ± 11	68 ± 11	66 ± 12	67 ± 14	72 ± 14	70 ± 11	58 ± 8	57 ± 7
Post-PET diagnostic confidence (%)	98 ± 4	70 ± 16	84 ± 17	83 ± 14	78 ± 13	76 ± 14	96 ± 5	79 ± 14
Δ Diagnostic confidence	25 ± 11^a^	1 ± 14	19 ± 18^a^	16 ± 16^a^	6 ± 15	6 ± 13	38 ± 10^a^	22 ± 16^a^
Increase in diagnostic confidence (%)	109 (99%)	17 (50%)	6 (100%)	18 (82%)	6 (75%)	7 (64%)	9 (100%)	11 (100%)

Data are presented as mean ± SD or *n* (%). Differences between pre-PET and post-PET diagnostic confidence were assessed using paired-sample *t* tests and presented as Δ diagnostic confidence

^a^Increased diagnostic confidence after PET, *P* < 0.05

*PET* positron emission tomography, *AD* Alzheimer’s disease dementia, *FTD* frontotemporal dementia, *OD* other dementia diagnosis, *NN* non-neurodegenerative diagnosis, *VaD* vascular dementia *DLB* dementia with Lewy bodies, *CBD* corticobasal degeneration, *CTE* chronic traumatic encephalopathy

In four out of six (67%) FTD patients with a positive PET scan, diagnosis changed to AD. In 2 out of 18 (11%) OD patients, a positive PET scan changed the initial diagnosis after PET, from CBD to AD and from PSP to DLB, respectively. In patients with an NN diagnosis and a positive PET scan (*n* = 9; one patient with OSAS, six patients with psychiatric disorders, two patients with meningioma), the post-PET diagnosis consequently changed into AD, which was the pre-PET differential diagnosis in all cases.

### Diagnostic confidence

Diagnostic confidence prior to PET did not differ between diagnostic groups except for the NN group, which showed lower diagnostic confidence (57 ± 7%) compared with the other diagnostic groups (71 ± 12%, *P* < 0.05). Overall, diagnostic confidence increased from 69 ± 12% before to 88 ± 15% after PET results were disclosed (*P* < 0.01). Increase in diagnostic confidence was seen in 183 patients (87%). A decrease in diagnostic confidence after PET was found in 28 (13%) patients, from 71 ± 11% before to 62 ± 12% after PET. Decrease in confidence was most often found in patients with a pre-PET AD diagnosis which changed after PET.

**Fig. 1:**
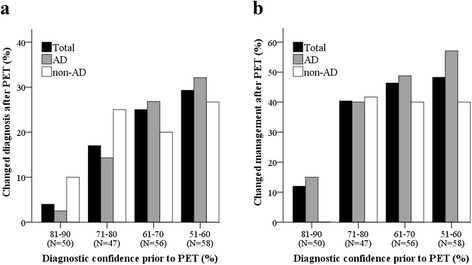
Diagnostic confidence prior to PET related to **a** changed diagnosis and **b** changed patient management plan. *AD* Alzheimer’s disease dementia, *non-AD* non-AD diagnosis, *PET* positron emission tomography

As presented in Table 2, an increase in diagnostic confidence after PET was found in all patients except for those with an initial AD diagnosis with negative PET results and OD patients. Diagnostic confidence prior to PET did not differ between diagnostic groups except for the NN patients, which showed lower diagnostic confidence (57 ± 7%, *P* < 0.05). In total, percent change in clinical diagnosis after PET increased with lower pre-PET diagnostic confidence (*P* < 0.01; Fig. 1a). This effect was found to be driven by AD patients (*P* < 0.01), because no association was found in non-AD patients.

### Patient management

PET results led to a change in the patient management plan for 79 out of 211 (37%) patients. The patient management plan altered more often for patients with a positive PET scan compared with those with negative PET results (42% vs 29%, *P* < 0.05).

Table 3 presents the impact of PET on the patient management plan according to clinical diagnosis prior to PET. Disclosing PET results led to a change in prescription of AD medication in 51 (24%) patients, which most often was the initiation of AD medication when the PET scan was found to be positive. Change in planned care was seen in 23 (11%) patients, which was independent of PET results. A change in the request for ancillary investigations was found in 22 (10%) patients. Overall, the clinician requested lumbar puncture results in 14 patients, additional [^18^F]FDG PET in eight patients, and [^11^C]PIB-PET in two patients (in both cases, [^18^F]flutemetamol and CSF results were contradictive). Eight patients were referred to a psychiatrist, one patient was referred to internal medicine, one patient underwent electroencephalography after sleep deprivation, one patient underwent dopamine transporter (DaT)-SPECT, and one patient underwent polysomnography.

**Table 3:** Impact of [^18^F]flutemetamol PET on patient management according to clinical diagnosis prior to PET

Pre-PET etiology	AD (*n* = 145)		FTD (*n* = 28)		OD (*n* = 19)		NN (*n* = 20)	
PET result	Positive	Negative	Positive	Negative	Positive	Negative	Positive	Negative
*n*	111	34	6	22	8	11	9	11
AD medication	39 (35%)^a^	1 (3%)	3 (50%)^a^	0 (0%)	1 (13%)	0 (0%)	6 (67%)^a^	1 (9%)
Care	12 (11%)	1 (3%)	1 (17%)	1 (5%)	0 (0%)	0 (0%)	4 (44%)	3 (27%)
Ancillary investigations	0 (0%)	13 (38%)^b^	1 (17%)	3 (14%)	1 (13%)	2 (18%)	0 (0%)	2 (18%)

Data are presented as *n* (%). Differences between impact of positive and negative PET results were assessed using χ^2^ tests

^a^Positive PET > negative PET, *P* < 0.05

^b^Negative PET > positive PET, *P* < 0.05

*PET* positron emission tomography, *AD* Alzheimer’s disease dementia, *FTD* frontotemporal dementia, *OD* other dementia diagnosis, *NN* non-neurodegenerative diagnosis

Overall, percent change in the patient management plan after PET increased with lower pre-PET diagnostic confidence (*P* < 0.01; Fig. 1b), which was found to be driven by AD patients (*P* < 0.01) because no association was found in the non-AD patients.

## Discussion

The aim of the present study was to assess the diagnostic impact of [^18^F]flutemetamol PET in patients suspected of early-onset dementia, who were visiting a tertiary memory clinic. We found that, after a standardized clinical work-up, [^18^F]flutemetamol PET results led to changes in clinical diagnosis, increases in diagnostic confidence, and alteration of the initial patient management plan in a substantial number of patients.

This prospective study predominantly included patients suspected of early-onset AD. In almost a quarter of these patients, PET showed no evidence of amyloid pathology, comparable with proportions found in clinical pathological comparison studies [19–21]. In line with previous studies, PET results were most often in agreement with the initial clinical diagnosis and overall resulted in increased diagnostic confidence. Subsequently, in these patients PET results more often led to the initiation of AD medication, as reported previously [7]. These findings suggest an additive but primarily confirmatory role for amyloid PET as a diagnostic marker in patients suspected of early-onset AD.

The overall impact on diagnosis seems to be somewhat lower compared with prior studies, although these finding were highly variable, ranging from 9 to 73% [5–11]. Lower impact might be explained by selection of a different patient population or the more liberal method of patient selection in the present study (diagnostic certainty < 90%), because we found that patients with less diagnostic certainty prior to PET were more likely to have their diagnosis changed after PET.

Of major interest were patients with inconsistent PET results according to their pre-PET diagnosis. In patients with an AD diagnosis and negative amyloid PET, clinicians remained uncertain about the underlying etiology. This probably explains that predominantly for these cases the clinicians requested further investigations after amyloid PET, most often [^18^F]FDG PET, to seek evidence for an alternative (nonamyloid) cause of the dementia.

In both FTD patients and patients classified as ‘non-neurodegenerative disease’, AD was often part of the differential diagnosis and subsequently positive PET results frequently led to a change in diagnosis to AD and increased confidence in the post-PET diagnosis, and often led to prescription of symptomatic treatment. In contrast, in patients classified as ‘other dementia’ prior to PET, scan results did not increase overall diagnostic confidence and rarely led to a change in pre-PET diagnosis. A possible explanation for this finding could be that in these patients AD was less often considered as a differential diagnosis prior to PET. An alternative explanation lies in the composition of the ‘other dementia’ group, because almost half of the patients were diagnosed with DLB prior to PET. In these patients, neither a positive nor a negative PET scan changed the initial DLB diagnosis, because amyloid pathology is known to occur in half of the DLB patients [22] and to a lesser extent in other non-AD dementias [23]. In only a few AD patients who turned out to have a negative amyloid PET scan was the AD diagnosis maintained. This is an interesting subset of AD patients that may give us more insight in the various underlying neuropathologies of AD phenotypes and warrant further investigation in future studies [24].

Appropriate use criteria (AUC) for clinical use of amyloid PET were published [4]. The preamble states that the dementia expert must expect that determination of amyloid status would both increase the level of diagnostic confidence and alter the plan for patient management. The present study included a large memory clinic patient sample suspected of mild and early-onset dementia, in which uncertainty in diagnostic confidence remained after standardized work-up. These inclusion criteria generally align with the AUC. However, part of our patients showed no increase in diagnostic confidence but did have a changed diagnosis, consequently resulting in an altered plan for patient management. Thus even without increase in diagnostic confidence, patients may benefit from amyloid PET, implying a more liberal application of the AUC.

We used [^18^F]flutemetamol PET as a surrogate marker for brain amyloid deposition. Previous studies have shown high correlation between [^18^F]flutemetamol retention and neuropathology findings [25–27]. In the present study an amyloid-positive PET scan often supported or changed a diagnosis into AD, nevertheless amyloid pathology was present in a few patients diagnosed with another dementia and interpreted as mixed or copathology and not the primary cause of the clinical manifestation of dementia. Because of the clinician’s awareness of the patients age prior to PET, the a-priori probability of detecting amyloid pathology related to age was part of the diagnostic decision-making [28].

Future analysis in this ongoing study will involve diagnostic accuracy of [^18^F]flutemetamol PET after a 2-year clinical follow-up period. Furthermore, health economic consequences for the use of amyloid PET in this setting are of great socioeconomic interest and will be assessed after clinical follow-up. In this respect, we would like to mention recent efforts to evaluate the effect of amyloid status disclosure, which showed a positive effect on caregivers [29].

Conducting a prospective study in a clinical cohort is accompanied by several limitations. First, investigations other than amyloid PET necessary for clinical diagnosis could have been ordered prior to PET (decisions that were not made based on PET results). More specifically, the lumbar puncture procedure is part of our standardized work-up. The results for amyloid 1–42, total tau, and p-tau, however, are not used during our multidisciplinary meeting when clinical diagnosis is made. The knowledge of availability of CSF biomarkers after post-PET diagnosis, however, may have had an effect on clinical decisions. This may have led to an underestimation of the impact on patient management in this study.

On the other hand, clinicians were aware of the fact that patients were included in the present study, which may have clinicians decide to postpone decision-making about patient management plan until PET results were disclosed, resulting in a relative overestimation.

Second, the vast majority of patients were included at the VUmc Alzheimer Center, which is a tertiary referral center with a high proportion of young patients with complex clinical presentations. The results of the present study are therefore probably not an accurate reflection of the effect of the use of amyloid PET in a general, often older aged, memory clinic population. Instead, these results support the notion in the appropriate use criteria for amyloid PET, describing a potential added value of amyloid PET in patients with early-onset dementia with unclear clinical presentation.

## Conclusions

Findings from this study indicate that [^18^F]flutemetamol PET has additive value in addition to standardized work-up in patients suspected of early-onset dementia, because it has an effect on clinical diagnosis, increases overall diagnostic confidence, and alters the patient management plan in over a third of patients. This study provides support for the recommendations put forward in the AUC for amyloid PET in clinical practice. Future research should focus on cost-effectiveness and patient experience for the implementation of amyloid PET in clinical practice.

## Abbreviations

AD: Alzheimer’s disease
AUC: Appropriate use criteria
bvFTD: Behavioral-variant frontotemporal dementia
CBS: Corticobasal syndrome
CDR: Clinical dementia rating
CSF: Cerebrospinal fluid
CTE: Chronic traumatic encephalopathy
DLB: Dementia with Lewy bodies
FDG: Fluorodeoxyglucose
FTD: Frontotemporal dementia
LP: Lumbar puncture
lv-PPA: Logopenic-variant primary progressive aphasia
MCI: Mild cognitive impairment
MMSE: Mini Mental State Examination
MRI: Magnetic resonance imaging
PNFA: Primary nonfluent aphasia
NN: Non-neurodegenerative diagnosis
OD: Other dementia diagnosis
OSAS: Obstructive sleep apnea syndrome
PET: Positron emission tomography
PSP: Progressive supranuclear palsy
SD: Semantic dementia
VaD: Vascular dementia
